# The gut bacterium *Extibacter muris* produces secondary bile acids and influences liver physiology in gnotobiotic mice

**DOI:** 10.1080/19490976.2020.1854008

**Published:** 2020-12-31

**Authors:** Theresa Streidl, Isabel Karkossa, Rafael R. Segura Muñoz, Claudia Eberl, Alex Zaufel, Johannes Plagge, Robert Schmaltz, Kristin Schubert, Marijana Basic, Kai Markus Schneider, Mamdouh Afify, Christian Trautwein, René Tolba, Bärbel Stecher, Heidi L. Doden, Jason M. Ridlon, Josef Ecker, Tarek Moustafa, Martin von Bergen, Amanda E. Ramer-Tait, Thomas Clavel

**Affiliations:** a Functional Microbiome Research Group, Institute of Medical Microbiology, University Hospital of RWTH, Aachen, Germany; b Department of Molecular Systems Biology, Helmholtz-Centre for Environmental Research, Leipzig, Germany; c Department of Food Science & Technology, University of Nebraska-Lincoln, NE, USA; d Max Von Pettenkofer Institute of Hygiene and Medical Microbiology, Ludwig-Maximilians-University of Munich, Munich, Germany; e Division of Gastroenterology and Hepatology, Department of Internal Medicine, Medical University, Graz, Austria; f Research Group Lipid Metabolism, ZIEL Institute for Food & Health, Technical University, Munich, Germany; g Institute for Laboratory Animal Science, Hannover Medical School, Hannover, Germany; h Department of Internal Medicine III, University Hospital of RWTH, Aachen, Germany; i Department of Microbiology, Perelman School of Medicine, University of Pennsylvania, Philadelphia, PA, USA; j Institute for Laboratory Animal Science, Faculty of Medicine, University Hospital of RWTH, Aachen, Germany; k Clinic for Cardiology (Internal Medicine I), University Hospital of RWTH, Aachen, Germany; l German Center for Infection Research (DZIF); Partner Site Munich, Munich, Germany; m Microbiome Metabolic Engineering Theme, Carl R. Woese Institute for Genomic Biology, Urbana, IL, USA; n Department of Animal Sciences, University of Illinois at Urbana-Champaign, Urbana, Illinois, USA; o Institute of Biochemistry, Leipzig University, Leipzig, Germany; p Nebraska Food for Health Center, University of Nebraska-Lincoln, Hannover, NE, USA

**Keywords:** Lipids, bile acids, gut microbiota, synthetic community, *Extibacter muris*, 7α-dehydroxylation, gut-liver axis

## Abstract

*Extibacter muris* is a newly described mouse gut bacterium which metabolizes cholic acid (CA) to deoxycholic acid (DCA) via 7α-dehydroxylation. Although bile acids influence metabolic and inflammatory responses, few *in vivo* models exist for studying their metabolism and impact on the host. Mice were colonized from birth with the simplified community Oligo-MM^12^ with or without *E. muris*. As the metabolism of bile acids is known to affect lipid homeostasis, mice were fed either a low- or high-fat diet for eight weeks before sampling and analyses targeting the gut and liver. Multiple Oligo-MM^12^ strains were capable of deconjugating primary bile acids *in vitro. E. muris* produced DCA from CA either as pure compound or in mouse bile. This production was inducible by CA *in vitro*. Ursodeoxycholic, chenodeoxycholic, and β-muricholic acid were not metabolized under the conditions tested. All gnotobiotic mice were stably colonized with *E. muris*, which showed higher relative abundances after HF diet feeding. The presence of *E. muris* had minor, diet-dependent effects on Oligo-MM^12^ communities. The secondary bile acids DCA and surprisingly LCA and their taurine conjugates were detected exclusively in *E. muris*-colonized mice. *E. muris* colonization did not influence body weight, white adipose tissue mass, liver histopathology, hepatic aspartate aminotransferase, or blood levels of cholesterol, insulin, and paralytic peptide (PP). However, proteomics revealed shifts in hepatic pathways involved in amino acid, glucose, lipid, energy, and drug metabolism in *E. muris*-colonized mice. Liver fatty acid composition was substantially altered by dietary fat but not by *E. muris.*In summary, *E. muris* stably colonized the gut of mice harboring a simplified community and produced secondary bile acids, which affected proteomes in the liver. This new gnotobiotic mouse model can now be used to study the pathophysiological role of secondary bile acids *in vivo*.

## Introduction

The gut microbiome, *i.e*. the community of microbes residing in the intestine of humans and other animals, plays an important role in host health and the development of chronic diseases such as metabolic disorders and liver cancer.^1,2^ In contrast to the high number of studies focusing on shifts in fecal microbiomes under disease conditions, there is a limited amount of data on how microbe-host interactions function, with few models available to study those interactions.^3,4^ Hence, there is an urgent need for experimental studies dissecting mediators of microbe-host interactions, such as the myriad of metabolites produced by microbes. Bile acids are among the best examples of such metabolites: they are synthesized in the liver in the form of conjugated primary bile acids that, once secreted in the small intestine, can be metabolized by gut bacteria. This bacterial metabolism substantially impacts the bioavailability and bioactivities of bile acids, which are known to regulate inflammatory and metabolic responses.^5^ Thus, it is essential to study the bacteria capable of converting bile acids.

The enzymatic reactions involved in bile acid metabolism by gut bacteria start with deconjugation (the removal of amino acid residues bound to primary bile acids) via bile salt hydrolase (BSH) activity.^6^ Free primary bile acids can then be metabolized further via removal of hydroxyl groups (dehydroxylation), oxidation (dehydrogenation), or epimerization.^7^ Whereas BSH activity is known to be carried out by a variety of phylogenetically diverse taxa,^8^ including species able to colonize the small intestine, the other reactions are thought to be restricted to bile acids escaping re-absorption in the ileum and catalyzed by a more restricted number of bacterial species colonizing distal parts of the gut. Whilst the so far best studied bile acid-dehydroxylating bacterium in the human gut is *Clostridium scindens*, species from mice are either unknown or, if already isolated, have not been taxonomically described and deposited in reference culture collections. One secondary bile acid-producing species that we have recently cultured and characterized from the mouse gut microbiota is *Extibacter muris*, the first representative of a novel genus which is able to metabolize the primary bile acids cholic acid (CA) to deoxycholic acid (DCA) via 7α-dehydroxylation.^9,10^

Considering the influence of bile acids on metabolic and inflammatory responses and the scarcity of experimental studies using bile acid-metabolizing gut bacteria,^11–16^ the goal of the present work was to assess the effects of targeted colonization by *E. muris in vivo*. To that end, germ-free mice were associated with the minimal bacterial community Oligo-MM^12,17^ with or without *E. muris* and fed one of two experimental diets varying in fat content. The main focus was to study responses in the liver because of its functional relevance for lipid metabolism.

## Results

### In vitro *metabolism of bile acids*

As a foundation for later *in vivo* experiments, we aimed to clarify the metabolism of bile acids by the Oligo-MM^12^ strains and *Extibacter muris in vitro*.

**Figure 1.**
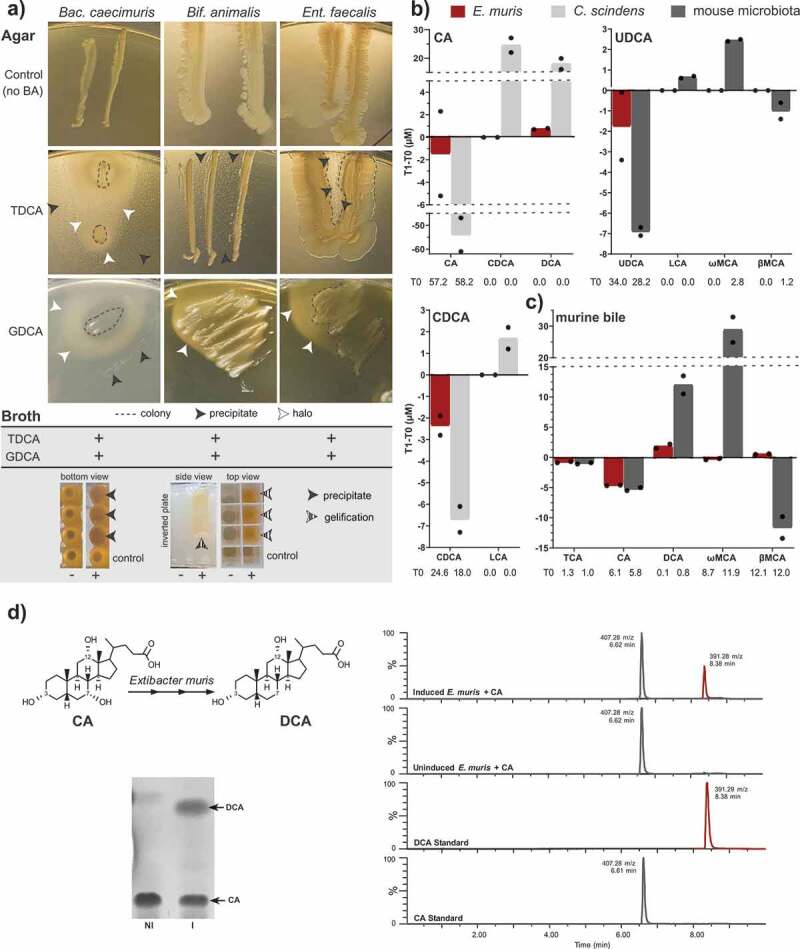
Bile acid transformation by OligoMM^12^ members and *E. muris* DSM 28560^T^. a) Deconjugation by the OligoMM^12^ strains *Bacteroides caecimuris* DSM 26085, *Bifidobacterium animalis* DSM 26074, and *Enterococcus faecalis* DSM 32036. The insoluble free bile acids released by deconjugation are visible as precipitates (black arrowheads) or halos (white arrowheads) surrounding the bacterial cell layer on agar plates (see images). In liquid medium, positive reactions (+) are recognized via the formation of precipitates at the bottom of culture wells (black arrowheads) and/or jellification of the medium (striped arrowheads), which remains in wells after inverting the culture plate. As negative controls (-), OligoMM^12^ strains were inoculated in medium without bile acids. b) Bile acids detectable by mass spectrometry before (T0) and after (T1) incubating *E. muris* for 48 h under anaerobic conditions with the respective bile acids, as indicated in the graphs. For the sake of clarity, delta-values (T1-T0) are presented on the y-axis; average concentrations at T0 are given below the x-axis for transparency. Incubations were performed in duplicates; single values are indicated with dots. c) Results of *E. muris* and complex microbial community incubated with mouse bile as described in detail in the methods section. The observed decrease in TCA was also observed in the negative control without bacteria and thus not due to *E. muris*, hinting at remnant BSH activity in the culture medium. The following bile acids were not detected in the respective incubation with CA: TCA, UDCA, LCA, ωMCA, βMCA; CDCA: TCA, CA, UDCA, DCA, ωMCA, βMCA; UDCA: TCA, CA, CDCA, DCA; and murine bile: UDCA, CDCA, LCA. d) Left-hand panel: Representative thin-layer chromatography depicting conversion of 100 µM CA by induced (I) or non-induced (NI) resting cells of *E. muris* DSM 28560^T^. Right-hand panel: Representative LC-MS chromatograms in single, negative ion monitoring mode overlaid with linked vertical axes of induced or non-induced *E. muris* reaction products compared to CA and DCA standards. Formula weight of CA is 408.57 atomic mass units (amu), DCA is 392.57 amu. Abbreviations: cholic acid, CA; deoxycholic acid, DCA

First, due to the release of bile acids by the host in conjugated form, Oligo-MM^12^ strains were tested for BSH activity via incubation with either TDCA or GDCA in both agar- and broth-based assays. Three Oligo-MM^12^ strains were consistently positive across the different assays: *Bacteroides caecimuris* DSM 26085, *Bifidobacterium animalis* DSM 26074, and *Enterococcus faecalis* DSM 32036 (Figure 1a). *Clostridium innocuum* DSM 26113 and *Flavonifractor plautii* DSM 26117 deconjugated TDCA but not GDCA, which tended to inhibit the growth of several strains under the conditions tested. *Muribaculum intestinale* DSM 28989 showed inconsistent results depending on the combination of bile acid tested and assay used: it was positive for TDCA on agar and GDCA in broth. *Lactobacillus reuteri* DSM 32035 was positive for GDCA deconjugation both on agar and in broth. The other Oligo-MM^12^ members either tested negative for all reactions or their growth was inhibited by addition of the bile acids (*Acutalibacter muris* DSM 26090, *Akkermansia muciniphila* DSM 26127, *Blautia coccoides* DSM 26115, *Enterocloster clostridioformis* DSM 26114, *Turicimonas muris* DSM 26109).

The previously observed ability of *E. muris* to produce DCA^10^ was confirmed, albeit at an amount approx. 24-fold lower than the positive control strain *C. scindens* (0.75 *vs.* 18 µM, respectively) (Figure 1b). The fact that the amount of DCA produced did not mirror the decrease in CA concentrations suggests the formation of intermediates (e.g. 3, 7, 12-oxo bile acids) not measured by the quantification method used. *E. muris* did not convert UDCA and CDCA into LCA when provided as single substrates under the conditions tested (Figure 1b). When incubated in the presence of mouse bile (1:300 dilution), *E. muris* produced up to 2.2 µM DCA, which was still ca. 6.5-fold lower than the complex mouse cecal microbiota used as positive control (Figure 1c). Moreover, *E. muris* was not able to catalyze the isomerization of βMCA into ωMCA.

We then tested whether bile acid metabolism is induced by CA in *E. muris* as observed in *C. scindens, Clostridium hiranonis*, and the closely related species *Clostridium hylemonae*, or if the activity is constitutive as observed in *Clostridium leptum* and *Clostridium sordellii*. Therefore, whole cell suspensions of *E. muris* that had previously been cultured in BHI medium with or without 100 µM CA were incubated for 8 h at 37°C in anaerobic PBS containing either 100 µM CA, UDCA, or β-MCA, followed by thin-layer chromatography (TLC) with confirmation by LC-MS. When CA was added to non-induced cells, very little to no DCA was visualized by TLC (Figure 1d). This result was corroborated by LC-MS: the primary reaction species eluted at 6.62 min with 407.28 m/z in negative ion mode, which is consistent with the elution time and formula weight (408.57 amu) of CA (Figure 1d). In contrast, CA incubation with induced cells of *E. muris* produced detectable levels of DCA via TLC and a corresponding peak of 391.28 m/z at 8.38 min that agreed with the mass and elution time of the DCA standard (392.57 amu). Incubation of UDCA or βMCA with induced or non-induced cells did not result in 7α-dehydroxylated products by TLC (data not shown).

Next, we identified known bile acid-metabolizing genes in the genome of *E. muris* DSM 28560^T^ by BLAST search against protein sequences from *C. hylemonae* DSM 15053^T^ (Supplemental Figure S1). We observed conserved baiBCDEFGHI polycistron and baiA1/3 monocistron preceded by conserved bile acid-inducible (bai) promoter between these two closely related species both able to 7α-dehydroxylate CA in an inducible manner. A partially conserved baiJKL operon was identified upstream of baiA, baiJ being a pseudogene in *E. muris* (Supplemental Figure S1). It was shown previously that baiK encodes a bile acid coenzyme A transferase,^18^ but the function of the other structural genes is not known. A gene reported to be involved in the reductive arm of the bile acid 7α-dehydroxylation pathway (baiN)^19^ was also conserved, as well as an NADP(H)-dependent bile acid 12α-hydroxysteroid dehydrogenase.^20^ These results are in line with the phylogenetic similarity between *E. muris* and *C. hylemonae*, indicate bile acid-inducible conversion of CA to DCA, but predict limited metabolism of UDCA and muricholic acids.

### *Stable colonization of* E. muris *in the intestine of OligoMM^12^ mice*

We then aimed to establish a gnotobiotic mouse model useful for studying the effects of secondary bile acid production on the host. Mice were therefore colonized with the simplified community Oligo-MM^12^ with or without the strain *E. muris* DSM 28560^T^ capable of dehydroxylating primary bile acids.^10^ Colonization was assessed in feces both in a non-targeted (high-throughput 16S rRNA gene amplicon sequencing) and targeted (qPCR) manner.

**Figure 2.**
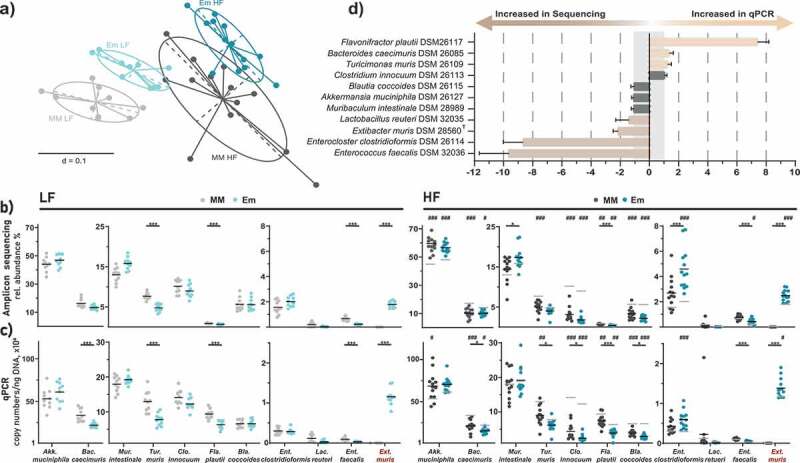
Stable colonization of *E. muris* in the intestine of OligoMM^12^ mice. a) *Beta*-diversity analysis shown as multidimensional scaling plot of generalized UniFrac distances (*p*-value <0.001; PERMANOVA). Bacterial composition in the gnotobiotic mice shown as b) relative abundances obtained by high-throughput 16S rRNA gene amplicon sequencing, and c) absolute gene copy numbers obtained by species-specific, 16S rRNA gene-targeted qPCR assays. d) Comparison of relative abundances between qPCR and 16S rRNA gene amplicon sequencing shown as ratios of respective relative abundances. For ratios below one, reciprocal values were calculated. Data are plotted as mean values ± SD. Significance is indicated as follows: one symbol, *p* < .05; two symbols, *p* < .01; three symbols, *p* < .001 (two-way ANOVA followed by Tukey test). Stars indicate differences due to *E. muris* (blue *vs*. gray). Hashtags indicate differences due to diet (mean values for LF diet-fed mice are shown again in gray in the plots for HF diet-fed mice)

After data processing, sequencing delivered an average of 30,962 ± 6,846 (Oligo-MM^12^ mice) and 32,168 ± 8,686 (*E. muris* mice) high-quality, chimera-checked, assembled sequences per sample. *Beta*-diversity analysis based on Generalized UniFrac distances revealed that the simplified communities were most distinct between the diets (low- *vs*. high-fat) with further separation according to colonization (presence or absence of *E. muris*), especially in the LF diet (Figure 2a).

Overall, ten of the twelve Oligo-MM^12^ strains stably colonized the mice (Figure 2b). The most dominant bacterium was *Akk. muciniphila* (>40% relative abundance in most mice), followed by *Bac. caecimuris* and *M. intestinale* (>10%), two strains able to deconjugate primary bile acids *in vitro* as presented above, *T. muris, C. innocuum, F. plautii* and *Bla. coccoides* (>5%). *Enterocloster clostridioformis, L. reuteri*, and *Enterococcus faecalis* occurred at lower relative abundances (<2%). *Bif. animalis* and *Acu. muris* were not detected, as reported earlier.^17,21^ *E. muris* colonized all target mice at an average relative abundance of approximately 1.8% during LF diet feeding and increased to 2.5% during HF feeding (*p* < .0001). These sequencing data were confirmed using qPCR (Figure 2c).

Depending on the diet fed and the methodology used for testing, colonization by *E. muris* triggered subtle changes in Oligo-MM^12^ composition (Figures 2b). Although the relative abundances (sequencing) and counts (qPCR) of *M. intestinale* and *Enterocloster clostridioformis* tended to increase in the presence of *E. muris* (especially under HF feeding), those of *Bac. caecimuris, T. muris, F. plautii* and *Enterococcus faecalis* decreased. With respect to diet, HF diet-fed mice were characterized by an increased occurrence of *Akk. muciniphila* and *Enterocloster clostridioformis* in addition to the aforementioned increase in *E. muris* when compared to the LF diet. The occurrence of *Bac. caecimuris, C. innocuum, T. muris*, and *Bla. coccoides* decreased during HF diet feeding (Figure 2b, c).

Comparison of the two microbiota analysis strategies (sequencing *vs*. qPCR) by calculating fold differences between strain compositions expressed as relative abundances (Figure 2d) showed consistent results for seven of the eleven bacteria detected. qPCR tended to overestimate the occurrence of *F. plautii* (7.4-fold) while sequencing favored the detection of *E. muris* (2.2-fold), *Enterocloster clostridioformis* (8.7-fold), and *Enterococcus faecalis* (9.7-fold).

### E. muris *triggered 7α-dehydroxylation and further rearrangements of bile acids composition* in vivo

Due to the ability of *E. muris* to dehydroxylate the primary bile acid CA *in vitro*,^10^ we tested whether successful colonization of gnotobiotic mice by this species affected bile acid pools in the cecum.

**Figure 3.**
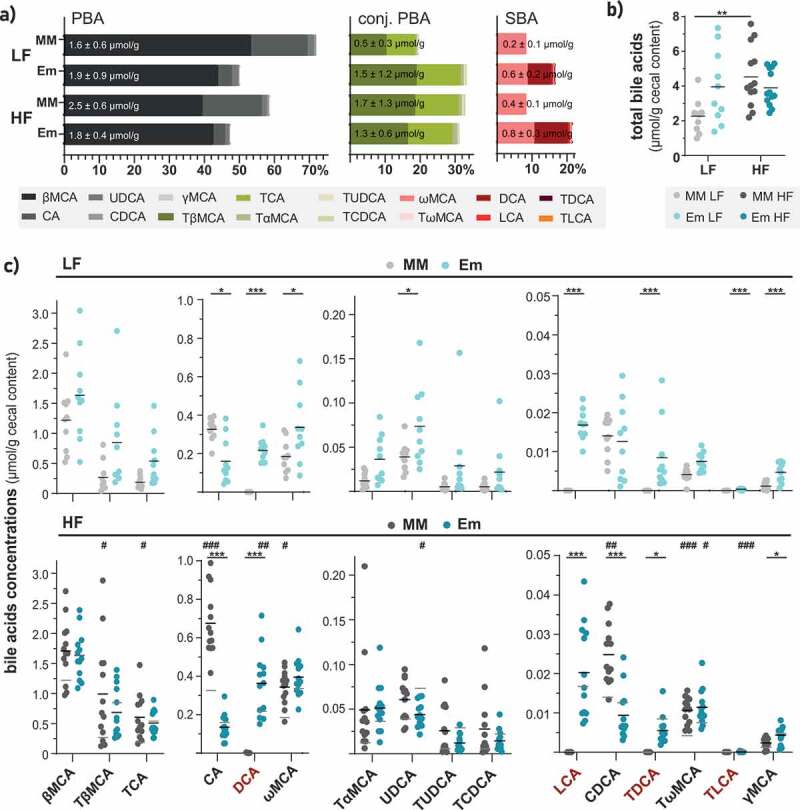
*E. muris* triggered 7α-dehydroxylation and further rearrangements of bile acids *in vivo*. a) Bile acid composition shown as percentages of total bile acids measured. The three main categories of bile acids considered were: PBA, free primary bile acids; conj. PBA, conjugated primary bile acids; SBA, secondary bile acids. Their respective average total amounts (in µmol/g cecal content, wet weight) are indicated in the corresponding stacked bar plots. b) Total bile acid concentrations. c) Concentrations of individual bile acids, shown in decreasing order: βMCA, β-muricholic acid; TβMCA, taurine-conjugated β-muricholic acid; TCA, taurine-conjugated cholic acid; CA, cholic acid; DCA, deoxycholic acid; ωMCA, omega-muricholic acid; TαMCA, taurine-conjugated α-muricholic acid; UDCA, ursodeoxycholic acid; TUDCA, taurine-conjugated ursodeoxycholic acid; TCDCA, taurine-conjugated chenodeoxycholic acid; LCA, lithocholic acid; CDCA, chenodeoxycholic acid; TDCA, taurine-conjugated deoxycholic acid; TωMCA, taurine-conjugated omega-muricholic acid; TLCA, taurine-conjugated lithocholic acid; γMCA, gamma-muricholic acid. Taurine-conjugated γMCA and glycine-conjugated bile acids were not detected. Data are shown and statistics performed as described in Figure 2

The secondary bile acids DCA and LCA and their taurine conjugates were detected exclusively in *E. muris*-colonized mice at a total concentration of 243 nmol/g during LF feeding, which represented ~7.5% of the total bile acids measured (Figure 3a). Under HF feeding, this fraction increased to 10.4% of total bile acids (*p* = .0744).

Although colonization by *E. muris* was associated with an increased total amount of bile acids when compared to the control OligoMM^12^ mice fed the LF diet (4.0 *vs*. 2.3 µmol/g; *p* = .0797), such an effect was not observed in mice fed the HF diet (3.9 *vs.* 4.5  µmol/g; *p* = .9997) (Figure 3b). In other words, HF feeding nearly doubled the total amount of bile acids in control OligoMM^12^ mice (*p* = .0055) while values were unchanged in HF diet-fed mice colonized with *E. muris*. The production of secondary bile acids in *E. muris*-colonized mice fed the LF diet was accompanied by a trend toward increased concentrations and proportions of conjugated primary bile acids (20 *vs*. 33%; *p* = .0561) (Figure 3a).

Regarding the concentration of individual bile acids, HF feeding increased mean cecal concentrations of DCA from 217 to 363 nmol/g (*p* = .039) in *E. muris*-colonized mice (Figure 3c; top *vs*. bottom panel; blue dots). In contrast, UDCA levels decreased in the same mice. The presence of *E. muris* led to reduced concentrations of the primary bile acids CA and CDCA, especially during HF feeding. Concentrations of ωMCA and the lowly abundant γMCA and UDCA were increased by *E. muris* colonization, albeit only with LF feeding.

### *Host responses to* E. muris *colonization*

Due to the successful production of secondary bile acids by *E. muris in vivo*, we next examined effects on the host. Many parameters such as liver histology, metabolic hormones, and inflammatory markers were significantly affected by HF diet feeding, but not by *E. muris* (Supplemental Figure S2). We subsequently investigated hepatic responses in greater detail using proteomics and quantitative lipidomics, as the liver is the primary site of energy and lipid metabolism.

**Figure 4.**
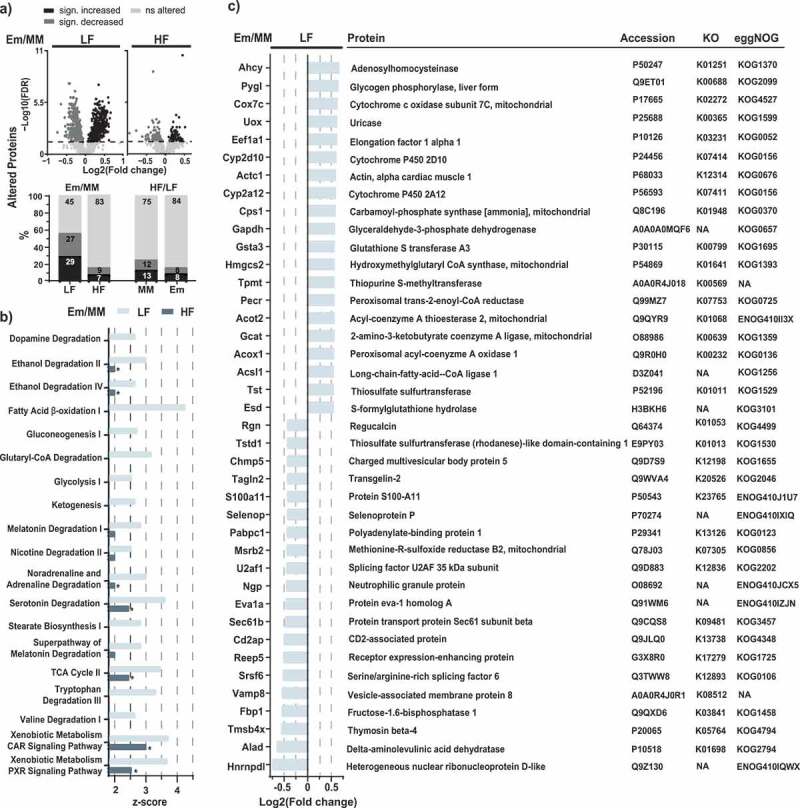
Liver proteomics. a) Volcano plots of proteins altered in mice colonized with *E. muris* compared to OligoMM^12^ in the two dietary treatments. The stacked bar plots below show the corresponding fractions of altered proteins, with alterations due to diet (HF *vs*. LF) as the comparison. b) Metabolic pathways significantly altered by *E. muris* in mice fed a LF diet (*p* < .05; z-scores > 2.5). Corresponding z-scores for mice fed a HF diet are shown, with stars indicating significance. c) List of top-20 most and least abundant proteins (see definition in the methods) in *E. muris*-colonized mice *vs*. Oligo-MM^12^ control receiving the LF diet. Proteins are ordered by fold-changes. Gene accession numbers, KEGG orthologues (KOs) and eggNOG accession numbers are indicated next to the protein names. NA, not available

A total of 1,158 ± 67 and 1,205 ± 81 reliably identified proteins were detected in mice fed the LF and HF diet, respectively. When comparing *E. muris* and OligoMM^12^ mice, a greater number of significantly affected proteins were found in mice fed the LF *vs*. HF diet: n = 682 *vs*. 224 proteins, with similar fractions of more and less abundant proteins (Figure 4a). Interestingly, *E. muris*-induced changes in the number of affected proteins were greater than those observed between HF *vs*. LF diets within colonization groups (MM, 334, 25%; Em, 209, 16%) (Figure 4a), suggesting enhanced hepatic functional changes in response to *E. muris* colonization rather than to dietary fat content.

Ingenuity Pathway Analysis (IPA) revealed that *E. muris* colonization affected 19 pathways, which were increased (z-scores > 2.5; *p* < .05) in mice fed an LF diet *vs*. 2 pathways in HF diet-fed mice. These 19 pathways were involved in amino acid, glucose, lipid, energy, and drug metabolism, and various degradation processes (Figure 4b). The most substantially affected pathways included those for fatty acid β-oxidation I (z-score of 4.2 for LF) and the xenobiotic metabolism CAR and PXR signaling pathways (z-score of both 3.7 for LF; 3 and 2.5, respectively, for HF). The overall lower z-scores for pathways altered by *E. muris* in mice fed the HF diet compared with the LF diet (Figure 4b) mirrored results observed at the level of protein numbers (Figure 4a). By extrapolation, those results are also consistent with the least total concentration of bile acids in OligoMM^12^ control mice fed the LF diet. In other words, the more pronounced effects of *E. muris* colonization in LF *vs*. HF diet-fed mice may result from a higher basal bile acid pool in OligoMM^12^ mice fed the latter diet, especially conjugated primary bile acids colonized with this species (Figure 3a). Several of the top-20 altered single proteins (see definition in the methods) in LF diet-fed mice belonged to the aforementioned pathways (Figure 4c). In particular, six of these proteins have been reported to interact with lipids or be linked to lipid metabolism: Hmgcs2,^22,23^ Eef1a1,^24,25^ Pecr,^26^ Acot2,^27^ Acox1,^28^ Acsl1,^29^ whereas some proteins were described to be bile acid-interacting proteins: Acox1, Acsl1, Sec61b, and Reep5.^30^

**Figure 5.**
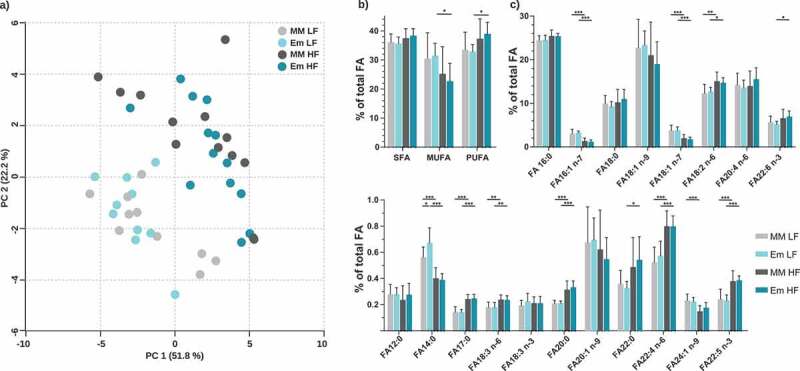
Total fatty acid analysis of liver samples. a) Principal component analysis (PCA) based on total FA profiles. b) Fatty acid distribution shown as percentages of total fatty acids quantified. Fatty acids categories were: SFA, saturated fatty acids; MUFA, monounsaturated fatty acids; and PUFA, polyunsaturated fatty acids. c) Total FA profile; fatty acids < 0.2% are not shown (C_10:0_, C_15:0_, C_20:4 n-3_, C_20:5 n-3_, C_22:1 n-9_). Data are plotted as mean values ± SD. *p < .05, **p < .01, ***p < .001 (two-way ANOVA followed by Tukey test)

Due to the observed alterations in hepatic pathways and proteins involved in lipid metabolism, total fatty acids were measured in liver samples by GC-MS (24 fatty acid species quantified). Neither dietary fat nor colonization by *E. muris* significantly affected the total amounts of fatty acids (LF MM, 130 ± 25; LF Em, 131 ± 17; HF MM, 175 ± 67; HF Em, 144 ± 37 nmol/mg; *p* > .05). Fatty acid profiles were distinct between diets, without further alterations due to the colonization status (Figure 5a). HF diet feeding shifted hepatic fatty acid compositions from monounsaturated to polyunsaturated fatty acids (Figure 5b), with increased proportions of C_18:2 n-6_, C_22:6 n-3_, C_18:3 n-6,_ C_22:4 n-6_, and C_22:5 n-3_ (Figure 5c). *E. muris* colonization slightly increased C_14:0_ fractions in LF diet-fed mice (0.56% *vs*. 0.67% total fatty acids; *p* < .05).

## Discussion

In the present work, we established a gnotobiotic mouse model that allowed studying the impact of secondary bile acids production on the host, in particular hepatic responses.

Multiple Oligo-MM^12^ members were able to deconjugate bile acids *in vitro*. This agrees with literature data on the overall widespread occurrence of bile salt hydrolases in phylogenetically diverse bacteria^6,31^ and strongly suggests that deconjugation is not a limiting reaction in our *in vivo* model. The fact that *Bac. caecimuris, Bif. animalis, Enterococcus faecalis, M. intestinale*, and *L. reuteri* tested positive *in vitro* is in line with a recent report on *bsh* gene prediction in the genome of these bacteria.^32^ However, no BSH-encoding genes were identified for *C. innocuum* and *F. plautii* in the latter study, although these Oligo-MM^12^ members tested positive for TDCA deconjugation in our experiments. This is possibly due to the high sequence homology between *bsh* and penicillin-V acylase genes^33^ and thus misleading genome annotations. Whilst it has been reported that BSH activity can be affected by the nature of conjugated amino acids,^34,35^ several OligoMM^12^ strains converted both tauro- and glycine-conjugated bile acids. We can only speculate about the fact that the growth inhibition by GDCA observed for several strains and the dominance of taurine conjugates in mouse bile are linked to functional adaptation of isolates originating from the mouse intestine.

*E. muris* DSM 28560^T^ stably colonized the intestine of C3H/HeN male mice. The presence of *E. muris* had only a minor impact on the occurrence of individual members of the reference simplified community OligoMM^12^ most likely due to their differing abilities to resist bile acids.^12,36,37^ The colonization data obtained by two different approaches (amplicon sequencing and qPCR) were altogether congruent: discrepancies were noted only for taxa detected at a low relative abundance by one of the methods, perhaps due to different primer binding and amplification efficacies.^38–40^ The occurrence of gut bacteria capable of dehydroxylating primary bile acids was previously reported to be increased during HF diet feeding,^41–43^ which was also observed specifically for *E. muris* in the present study. The occurrence of the OligoMM^12^ species *Akk. muciniphila* was also substantially increased by the HF diet, possibly due to the ability of this species to turn toward mucin degradation when less carbohydrates are available from the diet.^44^ However, several studies have previously reported a lower occurrence of *Akk. muciniphila* in mice fed HF diets.^45–47^ It should be noted that basal levels of this species in our control mice appeared higher than in previous OligoMM^12^ colonization studies performed in other mouse facilities.^12,17,21,48^ This most likely occurred due to the use of an experimental diet and not a standard chow diet as the control (relative abundances of *Akk. muciniphila* in the gut of our breeder mice fed a chow diet were approx. 25 ± 5 as analyzed by amplicon sequencing). In contrast to the previously reported bloom in *Erysipelotrichaceae* under high-fat diet feeding,^49,50^ the occurrence of *C. innocuum*, a member of this family,^51^ decreased in the present experiment. Altogether, it seems that HF-diet induced changes in gut bacterial populations using simplified communities does not necessarily mirror results obtained in conventionally colonized mice, although other confounding factors such as detailed diet composition, duration of feeding, or mouse genetic background certainly play a role too.

In addition to successful colonization, *E. muris* was able to catalyze 7α-dehydroxylation of bile acids *in vivo*. Although it did not produce LCA from CDCA *in vitro* in contrast to previous reports on its closest phylogenetic *Clostridium hylemonae* ANI 82.6,^10,11^ both *E. muris* and *C. hylemonae* appear to be incapable of converting UDCA *in vitro*.^52^ However, LCA was detected in *E. muris*-colonized mice, albeit at low concentrations (ca. 17 nmol/g cecal content on average). The *in vitro* conditions may have been unfavorable to LCA production by *E. muris*, even though experiments were performed in two different laboratories and also under induction by CA. Further work will be necessary in order to resolve this discrepancy; mouse-derived and other environmental factors may be required for LCA production by *E. muris in vivo*. The absence of murideoxycholic acid (MDCA) in *E. muris*-colonized mice corroborated the absence of MCA conversion *in vitro*. To the best of our knowledge, there is currently no isolate from the mouse gut known to metabolize the dehydroxylation of MCAs as observed previously in rats,^53^ stressing the need to continue cultivating and characterizing intestinal bacteria. The fact that cecal concentrations of ωMCA and γMCA increased in the presence of *E. muris* (especially in the control diet) may thus be linked to indirect effect via the host or OligoMM^12^ members. The trend toward increased concentrations of total bile acids due to *E. muris* colonization during LF diet feeding was accompanied by higher levels of the FXR antagonist TβMCA and lower concentrations of the FXR agonists CDCA and CA, which may have contributed to reducing the negative feedback loop for bile acid biosynthesis.^54,55^ This conclusion is further supported by an increase in the pathway “bile acid biosynthesis neutral pathway” (z-score 2.2) solely in mice fed an LF diet. HF diet feeding triggered an increase in total bile acids as described in the literature,^56^ without further changes due to *E. muris*.

Very few studies have investigated the impact of targeted colonization of mice with 7α-dehydroxylating bacteria; those that have all used strains originating from the human gut. Narushima *et al*.^13–15^ performed a series of experiments in gnotobiotic mice. In 1999, they showed that *Clostridium hiranonis* JCM 10541^T^, JCM 10542, and *Eggerthella lenta*-like strain c-25 (formerly referred to as *Eubacterium lentum*) were able to colonize mice at cell densities of 10^9^–10^10^ CFU/g feces after 3 weeks. In the presence of bile acid-deconjugating bacteria, no DCA was detected in mice colonized with *C. hiranonis* JCM 10541^T^, whereas DCA accounted for approx. 1% and 4% of total bile acids in cecal contents of mice colonized with *C. hiranonis* JCM 10542 and *E. lenta*-like strain c-25, respectively.^14,15^ Of note, recent extensive investigations of two *E. lenta* strains did not report 7α-dehydroxylating enzymes in this species,^57^ suggesting either strain-level differences or misleading identification of the aforementioned strain c-25 according to morphological and growth parameters only.^58^ In another study by Narushima *et al*.^13^ colonization by different bile acid-deconjugating strains in combination with *Clostridium hiranonis* JCM 10541^T^ or with five phylogenetically related strains at a total density of <10^10^ CFU/g feces was associated with the detection of 26–38% and 23–30% DCA (percentage of total bile acids), respectively. Three recent studies by Studer *et al*.^12^ and Marion *et al*.^16,32^ analyzed the impact of the human isolate *Clostridium scindens* ATCC 35704^T^ on bile acid metabolism in OligoMM^12^ mice. Colonization was accompanied by DCA production at ca. 0.08 to 0.34 µmol/g and by up to 0.14 µmol/g LCA. Ridlon *et al*.^11^ recently studied the B4PC2 synthetic gut community, which includes the human isolates *C. hylemonae* DSM 15053^T^ and *C. hiranonis* DSM 13275^T^. These bacteria colonized mice at mean relative abundances of 0.41% and 0.14%, respectively, which was linked to concentrations of 0.36 µmol DCA/g dried cecal content and 0.2 µmol/g LCA after 27 days. Due to different designs (*e.g*. mouse genetic backgrounds, diets) and methodologies (*e.g*. bile acid quantification, bacterial detection methods), comprehensive comparison of these models with the results obtained here is not possible. Altogether, our findings show that the mouse gut bacterium *E. muris* colonized the intestine of gnotobiotic mice reproducibly and at densities higher than human isolates of 7α-dehydroxylating bacteria. The stable colonization of *E. muris* was even vertically transmitted, which has not yet been described for 7α-dehydroxlating bacteria. Nonetheless, levels of the secondary bile acids DCA and LCA produced by *E. muris* correspond to concentrations observed in the models described by Studer *et al*.^12^ and Ridlon *et al*.^11^

The production of secondary bile acids in *E. muris*-colonized mice fed both diets was associated with marked activation of the pathway xenobiotic metabolism via both CAR and PXR signaling. LCA can either directly^59^ or indirectly^60^ activate both receptors, thereby leading to clearance of toxic bile acids, which also implicates energy metabolism via the repression of enzymes involved in gluconeogenesis and β-oxidation.^61,62^ However, such a repression of β-oxidation contradicts the observed *E. muris*-driven activation of this pathway in our experiments (LF diet-fed mice only), although bile acids are known to regulate lipid metabolism at multiple levels.^30,63^ Despite the tendency toward higher levels of secondary bile acids in *E. muris*-colonized mice fed the HF *vs*. LF diet, we observed fewer differences in liver proteomes between colonization groups fed the HF diet. This may be related to an already substantial rearrangement of metabolic pathways due to the diet itself,^64^ as exemplified by the observed higher concentrations of taurine-conjugated primary bile acids (TMCA and TCA) in control OligoMM^12^ mice fed the HF diet.

The observed shifts in liver proteomes were not linked to pathological changes in anthropometric measurements, blood parameters, inflammatory cytokines, or hepatic dysfunction as indicated by increased serum aspartate aminotransferase levels,^65^ or histological changes such as inflammatory infiltration or cell death.^66,67^ Kuno *et al.^65^* suggested that liver toxicity is dependent on the concentration of secondary bile acids, with physiological levels actually contributing to homeostasis. The levels of DCA and LCA observed in *E. muris*-colonized mice are in the range of concentrations normally detected in SPF mice and can therefore be considered as physiological.^56,68^ The effects of colonization on lipid pathways detected by proteomics did not translate into alterations of hepatic fatty acid profiles. In contrast to experiments by others, HF diet feeding did not lead to significantly increased amounts of total fatty acids in the liver, perhaps because diets were only fed for 8 weeks.^69^ Shifts from MUFA- to PUFA-dominated profiles due to dietary fat (lard in this study) have not been described before. The observed high levels of the fatty acid C_22:6 n-3_ appear remarkable due to its absence in both diets, maybe due to increased activity of host-derived PUFA-metabolizing enzymes, such as fatty acid desaturases (FADS) 1 and 2, and elongases (ELOVL) 2 and 5.^70–72^

## Conclusion

This study reports *in vivo* data on the only mouse gut bacterium capable of catalyzing 7α-dehydroxylation of bile acids that is taxonomically described and publicly available to date. Stable colonization and production of secondary bile acids were demonstrated with no pathological consequences for mice. This work opens avenues for experimental investigation of the causal role of secondary bile acids in mouse models of metabolic and other noncommunicable diseases.

## Methods

### Bile salt hydrolase assays

All Oligo-MM^12^ strains were tested for BSH activity *in vitro* using agar- and broth-based assays as described elsewhere.^73–75^ All tests were performed at least in three independent experiments.

For agar assays, autoclaved brain-heart-infusion (BHI) medium containing 1.5% agar was allowed to cool down to ca. 50°C and then supplemented with: (i) filter-sterilized L-cysteine and 1,4-Dithiothreitol (DTT) at a final concentration of 0.05% (w/v) and 0.02%, respectively; (ii) taurodeoxycholic acid (TDCA) (Sigma Aldrich, cat. no. T0875) or glycodeoxycholid acid (GDCA) (Merck, cat. no. 361311) at a final concentration 0.5% (w/v). The plates were brought into the anaerobic chamber two days prior to inoculation from a single colony of each target strain. For liquid assays, the anoxic BHI medium (0.5 mL) as described above but without agar and supplemented with 1.5% (w/v) TDCA or GDCA was dispensed into the wells of a 96-deep well plate. Freshly grown cultures (1 mL) of each Oligo-MM^12^ strain were then added in triplicate into the medium prior to incubation.

In all assays, the medium without bile acids was used as negative control for each Oligo-MM^12^ member. *Escherichia coli* DSM 106279 and *Fusobacterium mortiferum* DSM 108838 (www.dsmz.de/pibac) were used as negative and positive control bacteria, respectively. Plates were incubated at 37°C for 3 to 7 days prior to visual examination as follows: (i) on agar plates, the insoluble free bile acids generated by deconjugation are visible as precipitates or halos around colonies; (ii) in liquid cultures, positive reactions are recognized via the formation of precipitates at the bottom of wells and/or jellification of the medium, which remains in wells after inverting the culture plates.

### In vitro *conversion of bile acids*

For tests with single substrates, the anoxic BHI medium (see above) in Hungate tubes was supplemented with 50 µM of either cholic acid (CA) (Sigma Aldrich, cat. no. C1254), chenodeoxycholic acid (CDCA) (Sigma Aldrich, cat. no. C8261) or ursodeoxycholic acid (UDCA) (Cayman, cat. no. 15121). The medium was then inoculated 1:10 with a freshly grown culture of *E. muris*.

For tests with native bile acids, bile was collected from three donor mice undergoing bile-flow experiments over 30 min, pooled, diluted approx. 300-fold in anoxic BHI medium, and filter-sterilized. The medium (10 mL per assay) was then inoculated with ca. 1 mL of each of the BSH-expressing strains *Bif. animalis* DSM 26074, *Enterococcus faecalis* DSM 32036, and *Bac. caecimuris* DSM 26085. After 16 h of anaerobic incubation at 37°C to deconjugate the primary bile acids, 2.5 mL of the filter-sterilized supernatant was supplemented with 0.1 mL of 17-fold concentrated, anoxic BHI broth and with 0.28 mL of a freshly grown culture of *E. muris.*

All conditions were tested in duplicate. A volume of 500 µl culture was collected before incubation (T0) and after 48 h at 37°C under anaerobic conditions. Samples were centrifuged (11,000 g, 5 min) and the supernatants were stored at −80°C until measurement by mass spectrometry (see below). *Clostridium scindens* DSM 5676 ^T^ was used as positive control for incubations with CA and CDCA. Mouse cecal and large intestinal content diluted 300-fold in anoxic PBS was used as positive control for incubations with UDCA and mouse bile. For each bile acid tested and the mouse bile, one negative control without bacteria, *i.e*., with addition of sterile anoxic PBS instead of the bacterial inoculum, was included.

### Resting cell assays and thin-layer chromatography (TLC)

*E. muris* was cultured in BHI (10 mL) or BHI + 100 μM CA for three passages. At an OD_600nm_ of 0.3, cultures were centrifuged. The medium was discarded, and the pellet washed in 10 ml anaerobic PBS containing 1 g/L L-cysteine (PBSC). The pellets were then transferred to 10 ml PBSC containing 100 μM of either CA, UDCA, or β-MCA and incubated at 37°C for 8 h. Reactions were terminated by the addition of 750 μL 1 N HCl and bile acids extracted from the buffer with 2 volumes ethyl acetate. The organic fraction was concentrated under nitrogen, spotted on Bakerflex IB2-F TLC plates and resolved with toluene:dioxane:glacial acetic acid (75:20:2 v/v/v). Plates were sprayed with 10% wt/v phosphomolybdic acid in ethanol and charred 15 min at 100°C for visualization.

### *Liquid chromatography-mass spectrometry (LC-MS) of* in vitro *bile acids*

LC-MS was performed on the residue resulting from concentration of the organic phase from whole cell reactions using a Waters Acquity UPLC coupled with a Waters Synapt G2-Si ESI MS (Milford, MA). For LC, a Waters Cortecs UPLC C18 column (1.6 μm particle size, 2.1 mm x 50 mm) was used at a temperature of 40°C. The injection volume was 1 μL. Mobile phase A consisted of 95% water, 5% acetonitrile, and 0.1% formic acid. Mobile phase B consisted of 95% acetonitrile, 5% water, and 0.1% formic acid. The mobile phase gradient was as follows: 0 min 90% A and 10% B, 7.5 min 50% A and 50% B, 8.0 min 0% A and 100% B, 10 min 90% A and 10% B. The total run was 10 min with a flow rate of 10 μL/min. MS was performed in negative ion mode with a nebulizer gas pressure of 400°C and gas flow of 800 L/h. The capillary voltage was set at 2,000 V in negative mode. The Waters MassLynx software (Milford, MA) was used to analyze chromatographs and mass spectrometry data.

### Mouse experiments

**Figure 6.**
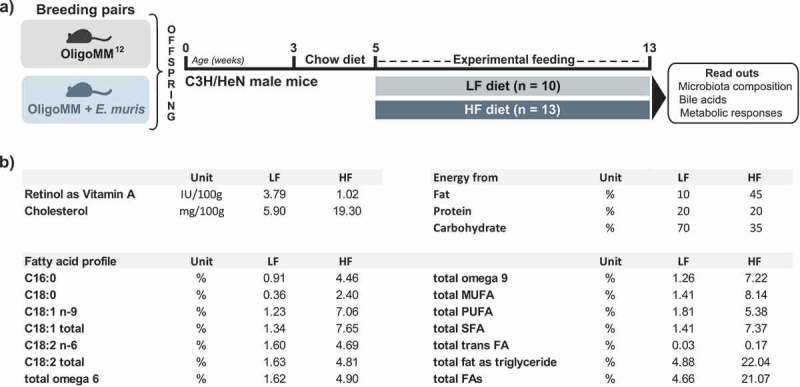
Experimental design and differential diet features. a) C3H/NeH male mice colonized from birth with the simplified community Oligo-MM^12^ with or without *E. muris* were fed two different diets from the age of 5 weeks on: (i) a control diet with low fat content (LF; 10% kcal from fat provided as lard; n = 10); (ii) a high-fat diet (HF; 45% kcal from fat; n = 13). The diets were fed *ad libitum* for a duration of 8 weeks. b) Major differences between the diets. All details are given in the methods section and in the Supplemental material. Abbreviations: MUFA, monounsaturated fatty acids; PUFA, polyunsaturated fatty acids; SFA, saturated fatty acids; FA, fatty acids

The experimental scheme is presented in Figure 6. Six- to eight-week-old germ-free C3H/HeN mice were colonized via oral gavage as previously described^76^ using snap-frozen cecal contents from Oligo-MM^12^ mice.^17^ Of note, whilst several of the Oligo-MM^12^ strains express bile salt hydrolases and can deconjugate primary bile acids, none of them is known to catalyze 7α-dehydroxylation. Mice were maintained in flexible film isolators under gnotobiotic conditions with a 14‐h light/10‐h dark cycle and controlled temperature and humidity at the University of Nebraska-Lincoln (USA).^76^ All mice were fed a standard chow (LabDiet 5K67, Purina Foods, St. Louis, MO). After confirmation of colonization with Oligo-MM^12^ (as tested in feces using 16S rRNA gene amplicon sequencing) and at least two generations of breeding, additional Oligo-MM^12^ mice were moved into a second isolator for colonization via oral gavage with 100 µL of a stationary phase culture of *E. muris* DSM 28560^T^ grown anaerobically in Wilkins-Chalgren broth supplemented with 0.05% (w/v) cysteine and 0.02% DTT. After confirmation of colonization with *E. muris* via 16S rRNA gene amplicon sequencing, colonies of gnotobiotic mice harboring either Oligo-MM^12^ or Oligo-MM^12^ plus *E. muris* were expanded via two generations of breeding to obtain 23 male offspring from each line (46 male mice in total) all born within two weeks of one another. At five weeks of age, the male offspring were moved to one of two experimental isolators: one for the Oligo-MM^12^ mice and another for the Oligo-MM^12^ plus *E. muris* mice (two or three mice per cage). At that time, mice were also assigned to one of two experimental diets prepared by Research Diets Inc. (New Brunswick, NJ, USA) and fed *ad libitum* for 8 weeks: (i) a control diet with fat from lard (LF; 10% kcal from fat and 1% sucrose, D12450K-1.5 V); (ii) a corresponding high-fat diet (HF; 45% kcal from lard and 18% sucrose, D12451-1.5 V). Diets were sterilized by irradiation (50 kGy) at Neutron Products (Dickerson, MD, USA). Major differences between the diets are summarized in Figure 6. Detailed lipid composition in the diets is provided in the Supplemental material 1. All procedures were approved by the Institutional Animal Care and Use Committee at the University of Nebraska-Lincoln (Protocols 817 and 1215).

### Sampling

Fresh fecal samples were collected from individual mice one week before necropsy (*i.e*. after 7 weeks of experimental feeding at the age of 12 weeks). At the age of 13 weeks, all mice were fasted for 6 h prior to sampling. Blood from the tail vein was used to directly measure fasting glucose (Accu-check, Aviva, Indianapolis, IN, USA) and to prepare serum for insulin measurement. Mice were then sacrificed via carbon dioxide asphyxiation. Blood was collected via cardiac puncture and mixed with either a combination of DPPIV inhibitor (Millipore, Billerica, MA, USA), a protease inhibitor cocktail (Sigma, Saint-Louis, MO, USA), and EDTA (Sarstedt, Nümbrecht, DE), or with EDTA only. After centrifugation (13,000 g, 4°C, 3 min), plasma was aliquoted and stored at −80°C until analysis. Organs were dissected, weighted and either immediately snap-frozen in liquid nitrogen or fixed in 4% paraformaldehyde for 48 h and then stored in 70% ethanol at room temperature. Epididymal, mesenteric and subcutaneous white adipose tissues (WAT) were weighed and the sum of all three tissues, hereon referred to as WAT mass, was calculated. Cecal contents were aliquoted into sterile Eppendorf tubes and immediately snap-frozen in liquid nitrogen. Frozen samples were stored at −80°C and shipped on dry ice when necessary.

### High-throughput 16S rRNA gene amplicon analysis

Fecal samples were processed as described previously.^77^ Briefly, metagenomic DNA was purified on columns after mechanical cell lysis. The V3/V4 regions of 16S rRNA genes were amplified (25 cycles) via a two-step PCR using primers 341 F and 785R^78^ following a combinatorial dual indexing strategy. An equimolar pool of the purified amplicon libraries was sequenced in paired-end mode on a MiSeq (Illumina) following the manufacturer’s instructions. Raw sequence reads were processed using the UPARSE-based^79^ platform IMNGS.^80^ Parameters were: barcode mismatches, 1; expected error, 3; quality trimming score, 20; trimming length, 15 nt; min. sequence length, 300 nt; max. sequence length, 500 nt. Operational taxonomic units (OTUs) were clustered at 97% sequence similarity and only those occurring at a relative abundance ≥0.25% in at least one sample across the entire dataset were kept for analysis.^81^ The identity of OTUs was determined using EZBioCloud.^82^

### Quantitative PCR of bacterial 16S rRNA genes

16S rRNA gene-targeted primers and hydrolysis probes for *E. muris* were designed using Primer Express 3 (Applied Biosystems, Life Technologies, USA): Forward: 5ʹ-TGATTACTAGGTGTCGGGAAGCA; Reverse: 5ʹ-CCCCAGGTGGATTACTTATTGC; Probe: 5ʹ-HEX-CTTCCCGGTGCCGCA-BHQ1. Primer specificity was confirmed against a mixture of OligoMM^12^ 16S rRNA gene-containing plasmids, for which signals were below the detection limit (Cq 30.5). Primers used for quantification of the OligoMM^12^ consortium were published elsewhere.^17^ Standard curves using 10-fold dilutions (n = 3 replicates each) of linearized plasmids containing the 16S rRNA gene sequence of either the individual Oligo-MM^12^ strains or *E. muris* were used as template DNA for the absolute quantification of 16S rRNA copy numbers. The efficiency of each qPCR reaction was calculated based on the slope of the standard curve. Quantitative PCR assays were performed as described previously.^17^

### Bile acid measurement

The protocol was published elsewhere.^83^ Briefly, frozen cecal contents (40–60 mg each) were sonicated for 30 seconds in methanol. Internal standards were then added. Analytes were separated by HPLC using a C18 reversed phase or pentafluorophenyl column prior to quantification by mass spectrometry. The following bile acids were measured: cholic acid (CA), ursodeoxycholic acid (UDCA), chenodeoxycholic acid (CDCA), deoxycholic acid (DCA), lithocholic acid (LCA), and their glycine and taurine conjugates; as well as αMCA, βMCA, γMCA, ωMCA, and their taurine conjugates.

### Plasma biochemistry measurements

Fasting insulin levels were measured from plasma originating from the tail vein using an Ultra-sensitive Mouse Insulin ELISA kit (Mercodia, Uppsala, SE). Levels of gut peptides, hormones and cytokines were measured in cardiac plasma using a Mouse Magnetic Bead Panel (Milliplex, Millipore, Billerica, MA, USA) and a MAGPIX instrument (Luminex Corporation, Austin, TX, USA). Aspartate aminotransferase (AST, ASTPNI: ACN 8680), cholesterol (CHO2I: ACN 8798 ID/MS standardization) and lactate dehydrogenase (LDH, LDHI2:ACN 8080) were measured in cardiac plasma at the central laboratory of clinical chemistry (University Hospital of RWTH Aachen). Of note, some sera were hemolytic, which may affect AST and LDH measurements.

### Liver histology

Formalin-fixed, paraffin-embedded liver samples were cut into 4-µm-thick sections using a Thermo Scientific HM 340E microtome (Fisher Scientific, Schwerte, DE), mounted on either Thermo Scientific SuperFrost Ultra Plus slides (Fisher Scientific, Schwerte, DE) or microscope slides (Carl Roth, Karlsruhe, DE), and heat-treated (37°C, overnight). The sections were stained with hematoxylin and eosin (HE) and covered with a glass cover slip before examination in a blinded manner with a Leica DM 2500 light microscope (Leica, Wetzlar, Germany) using a semi-quantitative scoring system (1, lowest; 5 highest). Evaluation criteria included degenerative changes, necrosis, hepatocytic vacuolization, fat accumulation, congestion, and cellular infiltration.

### Proteomics

Cold lysis buffer (40 mM Tris base, 7 M urea, 4% (w/v) CHAPS, 100 mM DTT, 0.5% (v/v) BioLyte) was added to 70–180 mg of frozen liver, followed by bead milling for 30–60 s and centrifugation (12,000 g, 5 min, 4°C). Benzonase (Merck, Germany) (3 µl) was added and the samples were incubated for 20 min at room temperature with subsequent centrifugation for 20 min at 12,000 g. The supernatant was recovered, and the procedure was repeated with the remaining pellet after adding 2 M thiourea in the lysis buffer. The supernatants were pooled and desalted on Vivacon 500 filter tubes (Sartorius, Germany) by applying them onto the filters followed by centrifugation (15 min, 14,000 g) and elution in 10 mM ammonium bicarbonate. Protein concentrations were determined using the Pierce 660 nm protein assay (Thermo Scientific, USA).

For untargeted proteomics, 50 µg protein was labeled with tandem mass tags (TMT-10-plex, Thermo Scientific, USA) as described in the manufacturer’s instructions.^84,85^ Labeled samples were analyzed as specified before^86,87^ on a nano-UPLC system (Ultimate 3000, Dionex, USA) equipped with a trapping column (Acclaim PepMap 100 C18, 3 µm, nanoViper, 75 µm × 5 cm, Thermo Fisher, Germany) and an analytical column (Acclaim PepMap 100 C18, 3 µm, nanoViper, 75 µm × 25 cm, Thermo Fisher, Germany) and coupled online to the mass spectrometer (QExactive HF, Thermo Scientific, USA) via a chip-based electrospray ionization source (Nanomate, Advion, USA). A non-linear gradient of 150 min was applied and the MS raw data were processed against the UniprotKB reference proteome of *Mus musculus* (6^th^ March 2019) using ProteomeDiscoverer 2.2. Protein fold-changes quantified in at least seven replicates were log2-transformed and median normalized before calculation of adjusted *p*-values (Benjamini-Hochberg method) and further analysis in R-3.5.0^88^ with the help of several packages: readxl, qpcR, plyr, splitstackshape, tidyr, calibrate, circlize, gplots, and ggplot2. Significantly altered proteins were ranked according to their fold change and the top-20 proteins were plotted. Ingenuity Pathway Analysis (IPA, Qiagen, Germany)^89,90^ was conducted for significantly altered proteins (adjusted *p*-value ≤ 0.05), with definition of mouse as organism and liver as tissue. All significantly altered pathways (independent of their z-score) and the top-20 altered proteins during HF diet feeding are available in the supplemental material 2.

### Total fatty acids analysis

Cold tissue extraction solution (MeOH:ddH_2_O 1:1, 1% SDS) was added to frozen liver samples (20–60 mg) in 2 ml screw cap tubes (Sarstedt, DE) prefilled with 0.7 g of ceramic beads (1.4 mm diameter, Bertin Technologies, FR) at a ratio of 1 ml per 50 mg. Samples were homogenized (30 sec, 6 m/s) using a FastPrep-24 homogenizer (MP Biomedicals). The tissue extracts (20 µl each) were then used for fatty acid derivatization. Fatty acid methyl esters (FAMEs) were generated by acetyl chloride and methanol treatment and extracted with hexane, as previously described.^91^ Total fatty acids were analyzed by gas chromatography coupled with mass spectrometry using a Shimadzu 2010 GC-MS system. FAMEs were separated on an SGE BPX70 column (10 m length, 0.10 mm diameter, 0.20 μm film thickness) using helium as carrier gas. The initial oven temperature of 50°C was increased to 155°C at 40°C/min, then to 210°C at 6°C/min, and finally to 250°C at 15°C/min. The fatty acid species and their positional and cis/trans isomers were characterized in scan mode and quantified by single ion monitoring to detect specific fragments of saturated and unsaturated fatty acids (saturated, m/z 74; mono-unsaturated, m/z 55; di-unsaturated, m/z 67; poly-unsaturated, m/z 79). The internal standard was non-naturally occurring C21:0 iso. Absolute fatty acid concentrations for all analyzed samples are available in the supplemental material 3. For principal component analysis (PCA) all fatty acids detected were considered.

### Statistics

Unless otherwise stated, data are presented as mean ± SD, and treatments were compared to that of sections scored ≥2 by Fisher’s exact test. Statistics for 16S rRNA gene amplicon data were compared by two-way ANOVA followed by Tukey test (*p < .05; **p < .01; ***p < .001). Graphs and statistics were generated using Prism (GraphPad, Version 8) and R (Version 3.6.1).^92^ For liver histology, sections rated as 1 were considered as healthy tissue and their prevalence was compared to that of sections scored ≥2 by Fisher’s exact test. Statistics for 16S rRNA gene amplicon data were calculated in R using Rhea.^93^ Statistical analyses of proteomics data were described above in the corresponding section. Principal component analyses of hepatic fatty acid compositions and host parameters were performed using MetaboAnalyst, including normalization by sum and autoscaling.^94^

## Supplementary Material

Supplemental Material

## Data Availability

Illumina reads of the 16S rRNA gene amplicon analysis are available at the NCBI SRA database under the accession number PRJNA633540. The proteomics data were deposited to the ProteomeXchange Consortium with the dataset identifier PXD019973 via the PRIDE partner repository.^95^ Raw lipidomics data will be made available by the corresponding authors upon reasonable request.
