# Supplementary material for: The gut bacterium Extibacter muris produces secondary bile acids and influences liver physiology in gnotobiotic mice
Source: Gut Microbes. 2020 Dec 31;13(1):1854008. doi: 10.1080/19490976.2020.1854008 (PMC7781625; doi:10.1080/19490976.2020.1854008)
Supplement: Supplemental Material [file KGMI_A_1854008_SM5090.zip › Supplementary information/Figure-S2_Host Parameters_200521_PCAwoOutliers.pdf]

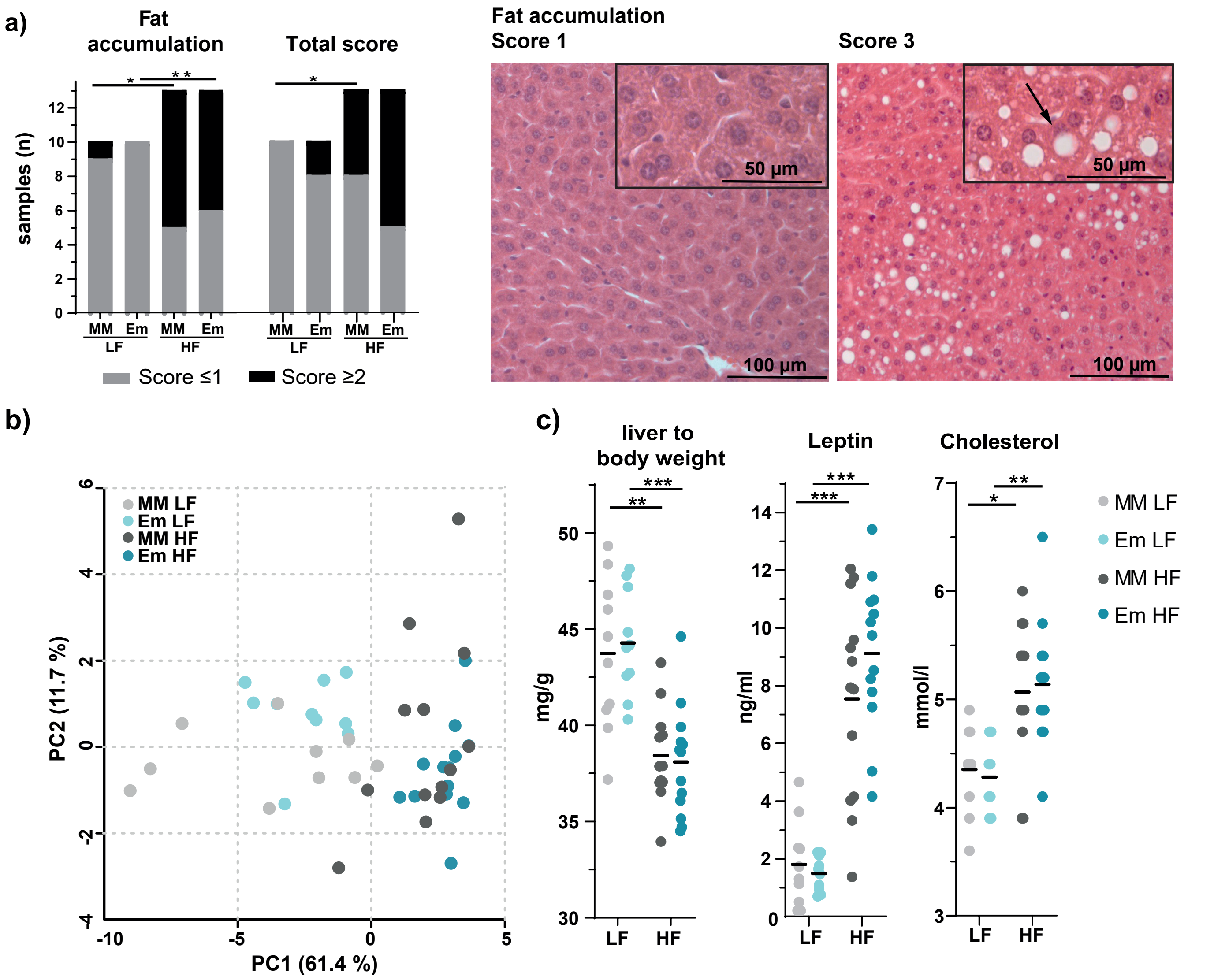

d)

		LF		HF	
	Unit	MM	Em	MM	Em
**AST**	U/l	182 ± 64	195 ± 85	183 ± 104	212 ± 102
**Amylin**	pg/ml	68.4 ± 36.1	75.3 ± 20.9	146.5 ± 76[a]	133 ± 66.9
**Body weight (BW)**	g	27.6 ± 1.9	27.1 ± 1.5	34.4 ± 2.9[a]	34.4 ± 1.5[a]
**Caecum weight**	g	0.35 ± 0.06	0.32 ± 0.05	0.23 ± 0.02[a]	0.21 ± 0.03[a]
**Ceacum/BW ratio**	mg/g	12.7 ± 2.5	11.7 ± 1.2	6.8 ± 0.7[a]	6 ± 1.1[a]
**C-Peptide**	pg/ml	1277 ± 385	1616 ± 384	2529 ± 1310[a]	2752 ± 711[a]
**GIP**	pg/ml	273 ± 123	280 ± 80	373 ± 90	469 ± 290
**Glucose (fasted)**	mg/dl	136.2 ± 8.6	130.7 ± 7.6	158.3 ± 11[a]	154.5 ± 20.9[a]
**Insulin (fasted)**	pg/ml	855 ± 468	1220 ± 489	2492 ± 1808[a]	2761 ± 1615
**LDH**	U/l	868 ± 283	849 ± 337	1036 ± 487	1138 ± 565
**Liver weight**	g	1.2 ± 0.1	1.2 ± 0.1	1.4 ± 0.2	1.3 ± 0.1
**MCP-1**	pg/ml	333 ± 45	256 ± 79	257 ± 52	260 ± 73
**PP**	pg/ml	51.8 ± 9.4	44.1 ± 10.1	44.9 ± 10.5	50 ± 13
**Resistin**	ng/ml	3843 ± 1520	3827 ± 832	6174 ± 1495[a]	8424 ± 2283[a,b]
**WAT mass**	g	1.4 ± 0.5	1.3 ± 0.3	3.2 ± 0.6[a]	3.4 ± 0.4[a]
