# Supplementary material for: The gut bacterium Extibacter muris produces secondary bile acids and influences liver physiology in gnotobiotic mice
Source: Gut Microbes. 2020 Dec 31;13(1):1854008. doi: 10.1080/19490976.2020.1854008 (PMC7781625; doi:10.1080/19490976.2020.1854008)
Supplement: Supplemental Material [file KGMI_A_1854008_SM5090.zip › Supplementary information/Supplementary figure legends.docx]

Figure S1. Conservation of genes involved in bile acid metabolism between *Extibacter muris* DSM 28560^T^ and *Clostridium hylemonae* DSM 15053^T^. Genes in *C. hylemonae* previously shown to be involved in bile acid metabolism were pBLAST searched against the genome of *E. muris* (NZ_SMMX00000000.1). Their organization and surrounding genomic context were compared. Percent amino acid sequence identity are provided for bile acid-metabolizing genes. Bile acid inducible (*bai*) promoters (P) are indicated and a biologos plot from Clustal Omega alignment of 54 bp upstream of the first codon is displayed.

Figure S2. Systemic blood, liver and body parameters. a) Histological evaluation of HE-stained liver sections rated for fat accumulation and total score comprising necrosis, hepatocytic vacuolization, fat accumulation, degenerative changes, congestion and cellular infiltration. Samples scored 1 were considered as healthy, whereas samples scored ≥2 were combined and their respective prevalence was plotted. Examples of stained tissue sections are shown for the lowest (1) and highest (3) possible scores. b) Diet-specific profiles revealed by principal component analysis (PCA); all host parameters were considered for analysis. c) Liver-to-body weight ratio, leptin and cholesterol levels in plasma. d) All other host parameters. Significances between dietary and colonization groups are marked with superscript letters (a and b, respectively). Data are shown as mean ± SD (n = 9-13). *p < 0.05, **p < 0.01, ***p < 0.001 (two-way ANOVA followed by Tukey test). Abbreviations: AST, aspartate aminotransferase; BW, body weight; GIP, gastric inhibitory polypeptide; LDH, lactate dehydrogenase; MCP-1, monocyte chemoattractant protein-1; PP, paralytic peptide; WAT, white adipose tissue mass.

Supplemental material 1. Detailed lipid composition in the diets.

Supplemental material 2. Significantly affected pathways and top-20 altered proteins during HF diet feeding, as analyzed via proteomics.

Supplemental material 3. Individual concentrations of fatty acids in liver samples.
