# Supplementary material for: The gut bacterium Extibacter muris produces secondary bile acids and influences liver physiology in gnotobiotic mice
Source: Gut Microbes. 2020 Dec 31;13(1):1854008. doi: 10.1080/19490976.2020.1854008 (PMC7781625; doi:10.1080/19490976.2020.1854008)
Supplement: Supplemental Material [file FigureS1_Extibacter genes_dinA4.pdf › Supplementary information/FigureS1_Extibacter genes_dinA4.pdf]

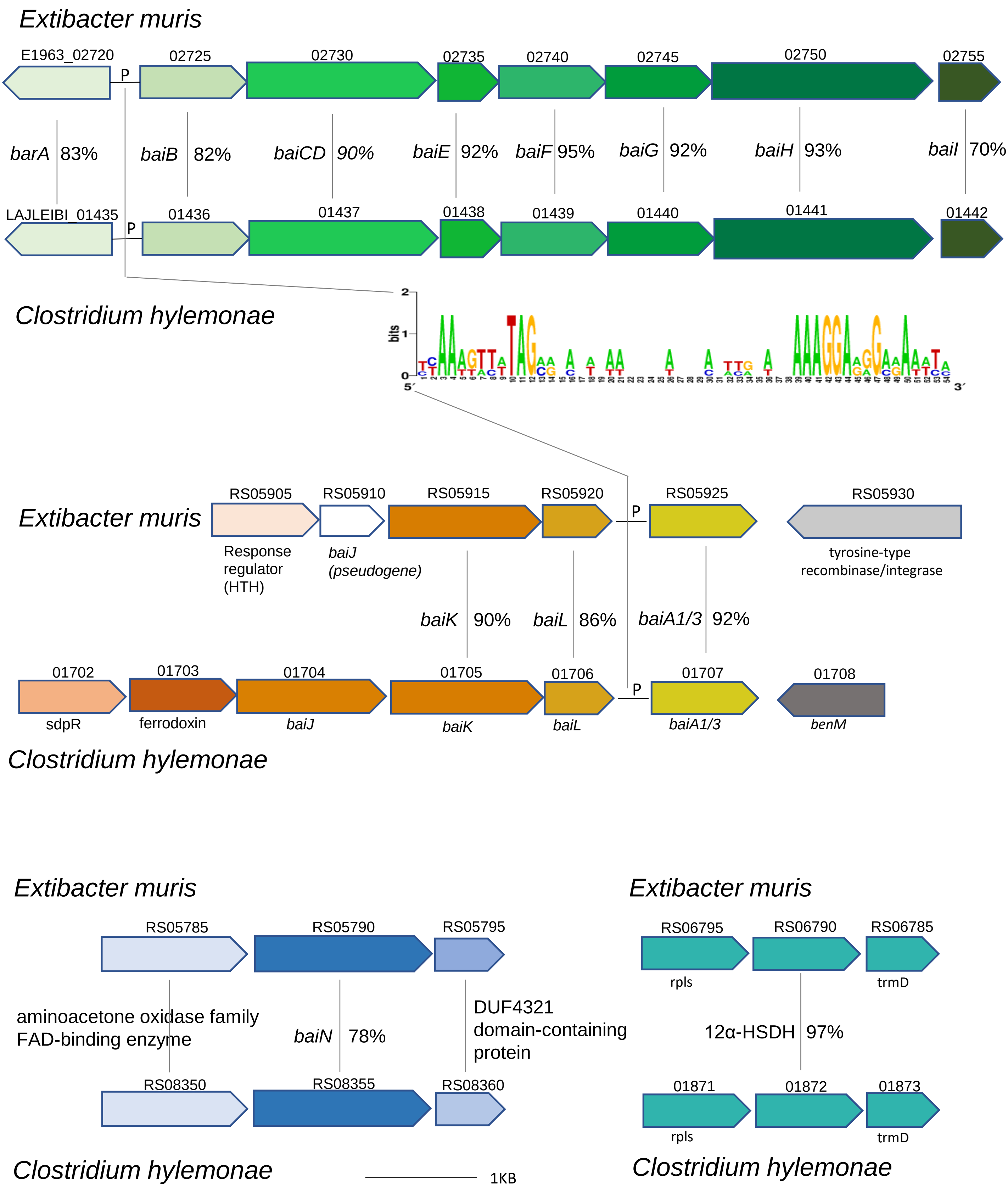

Extibacter muris
E1963_02720
P
02725
02730
02735
02740
02745
02750
02755
barA 83%
baiB 82%
baiCD 90%
baiE 92%
baiF 95%
baiG 92%
baiH 93%
baiI 70%
LAJLEIBI_01435
P
01436
01437
01438
01439
01440
01441
01442
Clostridium hylemonae
bits
5′
3′
Extibacter muris
RS05905
RS05910
RS05915
RS05920
P
RS05925
RS05930
Response regulator (HTH)
baiJ (pseudogene)
tyrosine-type recombinase/integrase
baiK 90%
baiL 86%
baiA1/3 92%
01702
01703
01704
01705
01706
P
01707
01708
sdpR
ferrodoxin
baiJ
baiK
baiL
baiA1/3
benM
Clostridium hylemonae
Extibacter muris
RS05785
RS05790
RS05795
aminoacetone oxidase family FAD-binding enzyme
baiN 78%
DUF4321 domain-containing protein
RS08350
RS08355
RS08360
Clostridium hylemonae
1KB
Extibacter muris
RS06795
RS06790
RS06785
rpls
trmD
12α-HSDH 97%
01871
01872
01873
rpls
trmD
Clostridium hylemonae
